# Supplementary material for: Apoptosis-Inducing Factor Deficiency Induces Tissue-Specific Alterations in Autophagy: Insights from a Preclinical Model of Mitochondrial Disease and Exercise Training Effects
Source: Antioxidants (Basel). 2022 Mar 7;11(3):510. doi: 10.3390/antiox11030510 (PMC8944439; doi:10.3390/antiox11030510)
Supplement: Supplementary file 1 [file antioxidants-11-00510-s001.zip › antioxidants-1586780-supplementary.pdf]

Supplementary Table S1. Antibodies used.

Primary antibody	Company Reference	Dilution (for WB)	Dilution (for IMF)
ACC1 (Ser79)	Abcam ab68191	1:5000	
26S proteasome	Abcam ab140675	1:1000	
AMPK (Thr172)	Cell Signalling #2535	1:1000	
AMPK total	Cell Signalling #5832	1:1000	
ATG16L	MBL PM040B	1:1000	
ATP5A	Abcam ab14748	1:5000	
Beclin-1	MBL PD017	1:1000	
Catepsina B	Cell Signalling #31718	1:1000	
GAPDH	SIGMA G9545	1:10000	
LAMP1	DSHB 1D4B	1:1000	
LC3	Sigma L7543	1:1000	1:500
mTOR (Ser2448)	Cell Signaling #5536	1:1000	
MYL3	ProteinTech 10913-1-AP	1:2000	
NDUFB8	Abcam ab110242	1:500	
p62	Abcam ab109012	1:10000	
TNNC1	ProteinTech 13504-1-AP	1:2000	
TNNI3	ProteinTech 21652-1-AP	1:2000	
TOM20	ProteinTech 11802-1-AP	1:10000	
γ-tubulin	BioRad VPA00655	1:2000	
VDAC1	Abcam ab14734	1:1000	

WB, western blot; IMF, immunofluorescence.
